# Vaccines against Group B Coxsackieviruses and Their Importance

**DOI:** 10.3390/vaccines11020274

**Published:** 2023-01-27

**Authors:** Kiruthiga Mone, Ninaad Lasrado, Meghna Sur, Jay Reddy

**Affiliations:** 1 School of Veterinary Medicine and Biomedical Sciences, University of Nebraska-Lincoln, Lincoln, NE 68583, USA; 2 Center for Virology and Vaccine Research, Beth Israel Deaconess Medical Center, Harvard Medical School, Boston, MA 02115, USA

**Keywords:** vaccine, coxsackieviruses, viral myocarditis, type 1 diabetes, viral insulitis

## Abstract

The group B coxsackieviruses (CVBs) exist in six serotypes (CVB1 to CVB6). Disease associations have been reported for most serotypes, and multiple serotypes can cause similar diseases. For example, CVB1, CVB3, and CVB5 are generally implicated in the causation of myocarditis, whereas CVB1 and CVB4 could accelerate the development of type 1 diabetes (T1D). Yet, no vaccines against these viruses are currently available. In this review, we have analyzed the attributes of experimentally tested vaccines and discussed their merits and demerits or limitations, as well as their impact in preventing infections, most importantly myocarditis and T1D.

## 1. Introduction

Enteroviruses belong to the *Picornaviridae* family, and *Homo sapiens* are the natural hosts of enteroviruses, which are known to cause a wide range of diseases [1,2]. In the current classification system, enteroviruses are sequentially numbered based on phenotypic and genetic similarity, and are classified into four distinct species: polioviruses, coxsackieviruses, echoviruses, and newly identified enteroviruses such as EV69, EV70, and EV71 [3,4]. While echoviruses affect the upper respiratory tract and central nervous system [5], coxsackieviruses can affect the cardiovascular, gastrointestinal, endocrine, neuromuscular, and cutaneous systems [6,7,8,9,10]. Coxsackieviruses are classified into coxsackievirus group A (CVA) and coxsackievirus group B (CVB) according to their organ tropism, organ damage, and antigenic response [11,12]. While CVAs are commonly implicated in the causation of hand, foot, and mouth disease [13], CVBs could induce diverse diseases. 

CVB was isolated for the first time at the Hudson River town of Coxsackie, New York, United States, in 1947 [14]. Six serotypes of CVBs (CVB1 to CVB6) have since been identified, and they have been associated with several diseases related to the heart, pancreas, brain, and gastrointestinal tract [15,16,17,18]. CVB1 is predominantly associated with type 1 diabetes (T1D) and can also cause pleurodynia, aseptic meningitis, and neonatal sepsis [15,19]. While CVB2 has been isolated from patients with acute myocarditis, aseptic meningitis, and acute meningoencephalitis [20,21,22], which can lead to multiorgan failure and cardiogenic shock [17], CVB3 infection is associated with myocarditis leading to dilated cardiomyopathy (DCM) and heart failure [23,24]. Likewise, CVB4 has been implicated in T1D development [16], while CVB5 is linked with conditions such as hand, foot, and mouth disease, aseptic meningitis, viral encephalitis, acute flaccid paralysis, myocarditis, and T1D [25,26,27]. However, isolated reports are available regarding the prevalence of CVB6 infection, but co-infection with CVB5 has been reported in patients with acute febrile illnesses, rash, severe acute respiratory disease, meningitis, myocarditis, and/or pericarditis [27]. Although all these infections are preventable, no vaccines against them are currently available, partly because it is impractical to develop serotype-specific vaccines. Furthermore, different CVB serotypes could affect different organs or they could induce similar diseases but with varied severities [28,29]. This complexity presents a challenge to determine which CVB serotypes should be considered in the vaccine design. 

## 2. Virus Infection and Disease Pathogenesis

CVBs, positive-sense single-stranded RNA (ssRNA) viruses, are generally considered lytic viruses, but they can persist in defective forms, as demonstrated with CVB3 [30]. They are small (30 nm), non-enveloped viruses, and the viral genome consists of approximately 7.4 kilobases (kb) with a single open reading frame flanked by 5′ and 3′ non-translated regions (NTRs) at the termini [31]. The viral genome lacks a 5′ cap structure, a typical feature of most eukaryotic and many positive-sense viral RNAs [32,33]. In the absence of a 5′cap, the 5′NTR accounts for 10% of the viral genome and contains an internal ribosome entry site that mediates the translation of positive-sense viral RNAs in infected cells [34,35,36].

CVBs are typically transmitted through the fecal–oral route. Infection of target cells requires interaction with two main receptors: the decay accelerating factor (DAF) and the coxsackievirus and adenovirus receptor (CAR) [37,38]; however, participation of other receptors, such as major histocompatibility complex I and heparan sulfate, may be critical [39]. The initial attachment with the DAF results in the rearrangement of cytoskeletal actin, making it easy for viruses to gain access to the CAR in tight junctions [11,38,40]. While all CVBs can bind, the CAR, CVB1, CVB3, and CVB5 could also bind the DAF. However, interaction with the DAF alone is insufficient to infect the target cells. Conversely, the binding of DAF-interactable CVBs in addition to binding with the CAR may facilitate a more effective infection [41,42,43,44]. This property may explain why CVBs could infect different organs since most hematopoietic and non-hematopoietic cells can express the DAF [45,46,47]. However, unlike the DAF receptor, the CAR exists in two isoforms (the seven exon-encoded CAR and the eight exon-encoded CAR) resulting from differential splicing [48,49]. The predominant form in humans is a transcript of ~6–6.5 kb (the eight exon-encoded CAR) expressed relatively highly in the heart, testis, prostate, and pancreas, as compared to the liver, brain, colon, and small intestine, but in mice, abundant expression of the CAR occurs in the liver, kidney, lung, and heart [50]. However, it is unclear whether the expression of one of the two isoforms described above is dispensable for infection; specifically dissecting this complexity may enable us to understand the selective infection of tissues by different CVB serotypes. Additionally, some CVB serotypes (CVB3 PD strain) could infect cells independent of the CAR and the DAF using the heparan sulfate [51,52], suggesting that alternative pathways could contribute to tissue specificity. 

Generally, the CAR acts as an internalization receptor for viral entry into the cytoplasm. After uncoating, the positive-sense ssRNA genome is translated, leading to the production of a single polyprotein followed by proteolytic cleavage, which generates structural viral proteins (VPs) 1 to 4 and nonstructural proteins namely, 2A^pro^, 2B, 2C, 3A, 3B, 3C^pro^, and 3D^pol^. While the structural proteins, also called capsid proteins, contribute to the assembly of the virus capsid, non-structural proteins are essential for viral replication and modulation of host responses [53,54,55,56]. The progeny of the virus is finally released through cell lysis. During the replication cycle, however, viral RNA could persist by forming a double-stranded RNA complex, as demonstrated with CVB1, but their ability to become infectious viral particles and their functions are unknown [53,54,57,58].

**Figure 1:**
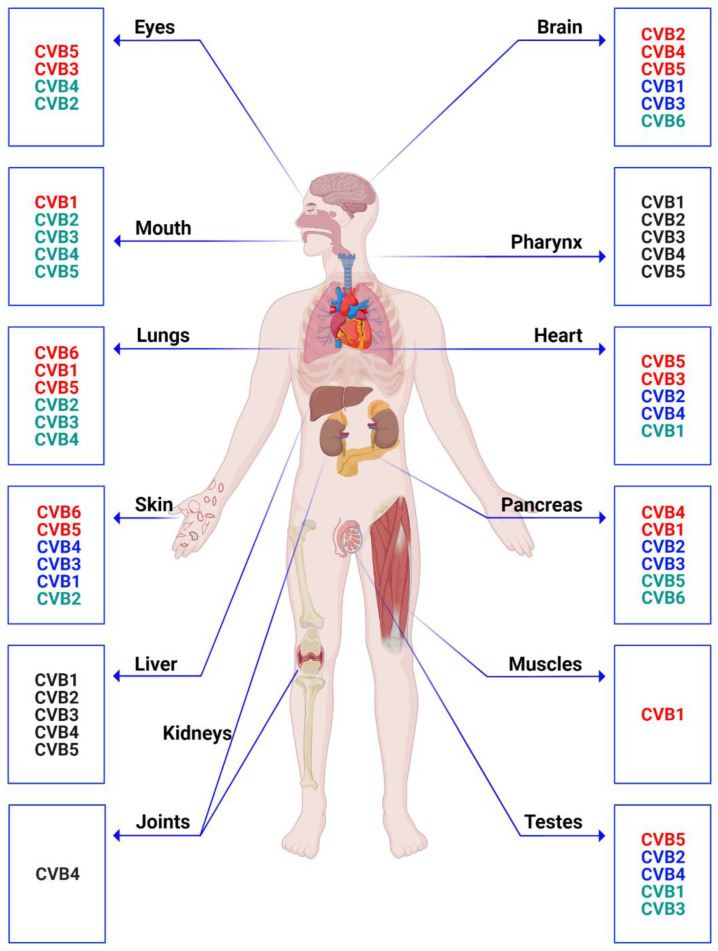
Organs affected by various serotypes of CVBs in humans. The serotypes affecting various organs are color-coded in each box. The red text indicates more commonly detected serotypes than others (blue and green) in that order, and the black text indicates no such differences. The information indicated in the figure was synthesized from Feigin and Cherry’s Textbook of Pediatric Infectious Diseases, 8th Edition 2019, and the figure was created using biorender.com.

Pathologically, both viral and host factors contribute to tissue damage in the affected organs [53,59,60]. Because CVBs are lytic viruses, tissue injury can result from apoptosis and necrosis of target cells. Additionally, nonstructural viral proteins can significantly alter the structure and functions of cellular proteins by various means, including shutting down of host proteins (eukaryotic initiation factor-4γ, Poly (A) binding protein, and cytoskeletal dystrophin) and cleavage of transcription factors (TATA-binding protein, octamer binding transcription factor, and cAMP response element binding protein), cell cycle arrest, and inhibition of vesicular transport [53]. Likewise, the host response to CVB infections contributes to inflammation and tissue destruction through the production of cytokines and lysis of infected cells by a variety of innate and adaptive immune cells that infiltrate the target organs [59,60]. It is unclear how different CVB serotypes cause different organ-specific diseases. For example, frequent disease associations have been made for almost all six CVB serotypes with respect to the heart, pancreas, brain, lungs, skin, eyes, and testes, followed by other organs such as muscles, the liver, pharynx, kidneys, and joints (Figure 1). Among these, the importance of CVBs in causing myocarditis and pancreatitis/insulitis has been better appreciated, as described below. 

Of the many types of heart disease, myocarditis is one predominant cause of heart failure in pediatric populations and young adolescents [61,62,63,64], and is the third leading cause of death in competitive athletes [65]. The global prevalence of myocarditis is estimated to range from 10.2 to 105.6 per 100,000 with an annual occurrence of ~1.8 million cases as of 2017 [66]. Most individuals affected with myocarditis remain asymptomatic, and the disease is spontaneously resolved. However, up to 30% of those affected can develop chronic disease leading to DCM, with an incidence of 3.5–8.5 cases per 100,000 population [61,67], and the highest numbers of mortalities resulting from cardiomyopathy/myocarditis were reported in the United States of America in 2019. Approximately half of DCM patients undergo heart transplantation due to a lack of chemotherapy options [68,69]. As to triggers, various infectious and non-infectious etiologies have been implicated in the causation of myocarditis, but the common suspects are viruses. In contrast to the earlier prevailing notion that enteroviruses and adenoviruses were found to be associated with viral myocarditis in North America and Europe, respectively, recent data suggest other viruses, such as human herpesvirus 6 and parvovirus B19, as well as coronaviruses [70], are increasingly being reported [71]. However, the major challenge is being able to demonstrate infectious virions in patients with myocarditis/DCM at the time of clinical presentation when viruses would have done the damage and be cleared from the bloodstream of affected patients. Thus, viral signatures may be the only readouts to determine their associations. In these scenarios, serologically, CVB-reactive antibodies are found in ~50% of DCM patients, while enterovirus genomic material can be detected in up to 70% [72,73,74,75,76,77,78,79,80,81,82], suggesting that CVB can be an important trigger of myocarditis/DCM, which may involve autoimmune responses to cardiac antigens [31]. 

Likewise, diabetes has become a major health concern with a global prevalence estimated at 537 million in 2021 with a projected increase to 643 million by 2030 (occurring in ~9% of adults) [83], and the incidence of T1D was 15 per 100,000 people with a prevalence of 9.5% [84]. The Centers for Disease Control estimates approximately 37 million Americans (~11.3%) are affected with diabetes, of whom nearly 1.9 million children and adolescents younger than 20 years have T1D. Among various types of viruses that may trigger T1D [60], two CVB serotypes (CVB1 and CVB4) are commonly linked with the development of T1D, as evidenced by serology, virus-recovery, and polymerase chain reaction analysis of viral genomic material from affected patients [85,86,87]. Additionally, T1D can be seen in families with no history of the occurrence of T1D [88,89]; type 2 diabetes (T2D) could coexist with T1D and T2D patients may have enterovirus levels higher than unaffected controls [90,91,92]. 

Since CVB infections are considered to be favored candidates in the occurrence of myocarditis/DCM and T1D, their prevention through vaccination can possibly lessen the incidence of both diseases. 

## 3. Current Status of CVB Vaccines

**Figure 2:**
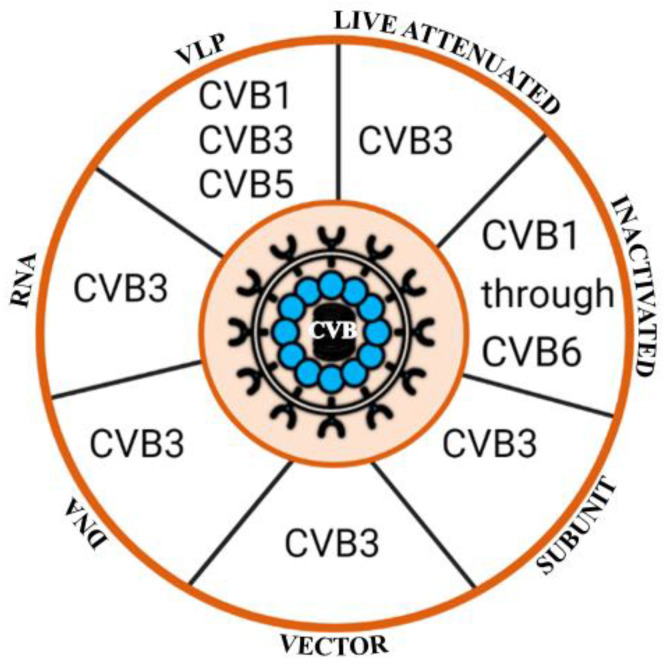
Experimental vaccines tested for various CVBs in the preclinical models. Several vaccine platforms shown with the upper-case text outside the big circle have been used to evaluate the efficacy of CVB vaccines in various preclinical models. While vaccines against CVB3 have been tested by using all the indicated approaches, killed vaccines have been tested for all six CVB serotypes, whereas VLP vaccines were tested only for CVB1, CVB3, and CVB5. The figure was created using biorender.com.

During the past two centuries, various vaccine platforms have been established, such as live-attenuated vaccines, inactivated vaccines, subunit vaccines, viral and bacterial vector vaccines, and nucleic acid vaccines, among others [93,94]. Many of these approaches have also been experimentally used to develop vaccines against CVB infections (Figure 2). Generally, the CVB3 infection model has been used in all platforms to test the efficacies of vaccines, likely because CVB3 infection consistently induces both myocarditis and pancreatitis in susceptible mouse strains. Thus, we first describe the characteristics of each vaccine design approach pertaining to CVB3, followed by other serotypes.

### 3.1. Modified Live-Attenuated Vaccines (MLVs)

The major advantage of MLVs is their ability to induce both humoral and cell-mediated immune (CMI) responses, especially cytotoxic T lymphocytes (CTLs), which are critical in eliminating established infections. MLVs for CVBs have been derived by either serially passaging the viruses in various cell lines or introducing mutations in the structural proteins, namely VP1 or VP2 or stem-loops II and V. As indicated in Table 1, the CVB3 vaccine strain termed p14V-1 was developed by attenuation after 14 serial passages in dermal fibroblasts (Table 1). In severe combined immunodeficiency disease mice, a mutation from aspartic acid [D] to glycine [G] in position 155 located near the viral canyon in the VP2 region, was shown to be responsible for the attenuated phenotype [95]. In subsequent studies, the p14V-1 was shown to be protected against CVB3-induced myocarditis and pancreatitis [96,97,98], and myosin-reactive antibodies were remarkably low [96]. This is a critical observation because CVB3 infection can lead to the de novo appearance of autoantibodies [59,99,100]. Additionally, we have demonstrated that CVB3 infection can lead to the generation of pathogenic cardiac-specific T cells by using major histocompatibility complex class II tetramers/dextramers [28,101]. Since cardiac antibodies were still detected in vaccinated (p14V-1) and challenged animals, it is possible that the vaccine recipients might have experienced some degree of heart damage. 

Likewise, the CVB3-RD strain, which was attenuated after serial passaging of the CVB3 Nancy strain in human rhabdomyosarcoma cells, had a mutation in the puff VP2 region (T151S), rendering the virus non-cardio virulent [102,103]. Similarly, the CVB3-RD strain was shown to be efficacious against CVB1 infection as a vaccine candidate, but pancreatic necrosis was still evident [104] (Table 1). Similarly, a series of attenuated strains, CVB3 (KR/EG/DE) and CVB3 (KR/EG/PM) were created by site-directed mutagenesis at D155E in the VP1 EF loop and P126M in the VP1 DE loop on a double mutant virus [CVB3 (KR/EG)] [105,106]. While studies with these mutants were shown to be non-cardiovirulent, small foci of pancreatic damage were still present in challenged mice (Table 1). Using the CVB3-H3 Woodruff strain, mutations were introduced by site-directed mutagenesis at Y240F, Y254F, and YYFF (both Y to F mutations at 240 and 245 together) in the C-terminal VP2 region of CVB3 [107]. The Y254F and YYFF mutations offered complete protection from CVB3-induced myocarditis and pancreatitis [107] (Table 1). Superior vaccine-induced neutralizing antibodies (nAbs) and CTL response, as noted with CVB3 (H3), suggest that MLVs bearing mutations in the C-terminal region may play an important role in viral pathogenesis. Such mutant viruses could structurally be defective but remain attenuated, leading to the induction of CTL responses. Other mutant viruses showing Sabin 3-like mutation in the stem-loop V (U475C) or mutations in the stem-loop II (88–186) were tested [106,108,109,110,111,112]. Although nAbs were noted against both CVB3 and CVB4, limited damage was noted in the myocardium and pancreas, especially by intraperitoneal route in the pancreas in the CVB3 Sabin 3-like virus/challenged animals. However, the animals administered with the clinical isolates with mutations in the stem-loop II (nt 88–186) showed low viral titers in the heart and pancreas and were protected against CVB3 infection [112]. These observations highlight the significance of point mutations in determining the virulence of viruses and their utility as vaccine candidates.

Several clinical isolates of CVB3 collected over the years were tested to determine the major nucleotide determinants of cardio virulence, which led to the identification of nucleotide 234 in the 5′NTR as the primary determinant of cardiac virulence of CVB3 [110,111]. Further studies revealed that the stem-loop II structure in the genome of the CVB3/GA isolate was responsible for virus clearance. Immunization with this mutant virus protected mice from CVB3-induced myocarditis and pancreatitis in challenge studies [113] (Table 1). Likewise, upon replacing 5′NTR from CVB3 with that of type 1 poliovirus, the newly generated CPV/49 virus induced nAbs and offered complete protection against both myocarditis and pancreatitis [114]. The protective effects of CPV/49 might be due to the finding that the 5′NTR of CVB3 is critical for viral positive-strand RNA translation and its absence may have caused the virus to lose virulence. 

Similar observations were noted with the temperature-sensitive mutants of CVB3 that replicate at 34 °C or 39.5 °C as vaccine candidates [115]. These mutants had defects in viral RNA synthesis, poor capsid stability, and virion assembly. Studies using temperature-sensitive mutants in CD-1 mice revealed protective effects through Immunoglobulin (Ig) G and IgM antibodies. The mice developed no myocarditis upon challenge, but interferon (IFN)-γ levels were low, indicating that the temperature-sensitive mutants may confer protection through antibody responses (Table 1) [116,117,118].

Attempts have been made to create a multivalent vaccine by creating an attenuated CVB3 encoding the L1 loop of the adenovirus (Ad2) hexon protein and the protease 2A protein, which induced nAbs against both CVB and Ads, but protective effects of the vaccine were not investigated [119]. Likewise, an approach to creating attenuated viruses by incorporating muscle-specific micro RNAs [miRNAs] (miRNA-133 and miRNA-206 into the 5′NTR has been described [120,121]). These attenuated viruses induced high nAbs and protected against CVB3 infection in challenge studies, although mild lesions were seen in the heart and pancreas. While such vaccines could potentially be used therapeutically and preventatively, off-target effects of micro RNAs miRNAs, if any, cannot be ruled out. Recently, we reported a mutant strain of CVB3 (Mt10), which we created by introducing the mutation in the CAR-binding region of the viral canyon within the VP1, expecting the attenuated virus to lose pathogenicity without affecting infectability, which is critical to induce CMI responses [123]. In addition to inducing nAbs against homologous (CVB3) and heterologous (CVB1 and CVB4) serotypes, the Mt10 vaccine protected completely against both myocarditis and pancreatitis induced by CVB3 in A/J mice [123]. More recently, we reported that the Mt10 virus could also offer significant protection (92%) against pancreatitis induced by CVB4 infection in A/J mice [122], and a similar level of protection (87%) was noted against CVB4-accelerated T1D in non-obese diabetic (NOD) mice [124]. The Mt10 vaccine virus was also found safe, as demonstrated by the absence of cardiac injury markers and cardiac-reactive T cells [123]. Although we have not tested the efficacy of the Mt10 vaccine against other CVB serotypes, the monovalent Mt10 vaccine virus has the potential to confer protection against multiple CVB serotypes through the generation of cross-reactive immune responses. In that direction, we are currently investigating the protective effects of the Mt10 virus vaccine in diversity outbred mice, whose genetic makeup has been derived from a combination of eight inbred mouse strains, including three wild-derived mouse strains [160]. Such preclinical studies in vaccine research are valuable since the observations made in the diversity of outbred mice may be relevant to the outbred human population, as testing in one inbred mouse strain is genetically akin to testing in a single person. 

Taken together, numerous approaches have been adopted to investigate the use of MLVs with mixed successes. Despite their ability to induce antibody and CMI responses, attenuated viruses can potentially revert to their virulence. Conversely, should the vaccine viruses retain mutations for a period of at least ~one month in vivo, protective immune responses would have been generated by then. Likewise, MLVs for CVB infections should be free of the autoimmune responses described above. These limitations can be overcome by developing inactivated/killed vaccines. 

### 3.2. Inactivated Vaccines

Inactivated vaccines have been traditionally prepared by killing the whole pathogen using formaldehyde, ultraviolet rays, X-rays, γ rays, β-Propiolactone (BPL), hydrogen peroxide, and zinc-finger reactive compound treatments, among others [161]. Although the killed vaccines do not revert to their pathogenic forms and are more stable, they tend to have reduced immunogenicity and require an adjuvant or multiple boosters to induce effective protective responses [161]. One of the first inactivated CVB3 vaccines was developed using BPL to inactivate CVB3 [125] (Table 1). Using Quil A matrix as an adjuvant, mice were immunized with BPL-inactivated CVB3 and later challenged with CVB3. The vaccine recipients had low nAb titers, with a survival rate of ~74% in challenge studies [125]. Subsequently, a polyvalent CVB1-6 formalin-inactivated vaccine was developed that gave protection against all six serotypes of CVB, but mild pancreatitis was still observed in some animals after the challenge [126]. Additionally, a three-dose regimen was needed to confer better protection [126], which also led to the induction of nAbs, but levels varied [126,127]. Further evaluation of this vaccine in New Zealand white rabbits revealed protection against all six CVB serotypes and induced nAbs against twelve clinical isolates. As to the prevention of T1D in the NOD model, formalin-inactivated CVB4-E2 delayed the onset of T1D, but autoantibodies to several Islet β cell autoantigens were elevated, potentially resulting from cross-reactivity [128]. More recently, another formalin-inactivated hexavalent vaccine was developed by incorporating all six CVB serotypes and tested against CVB1 and CVB4-induced T1D using CVB1 and CVB4 monovalent vaccines as positive controls [129,130,131,132,133] (Table 1). The vaccine-induced protection against multiple CVB serotypes in the Balb/c, NOD, and suppressor of cytokine signaling-1 (*SOCS-1*) transgenic mice models, preventing CVB3-induced myocarditis and pancreatitis with no mortalities, as well as preventing CVB1- and CVB4-induced T1D. The immunogenicity of the hexavalent vaccine was also recapitulated in non-human primates. The three-dose vaccine regimen eliciting virus-reactive Abs may have contributed to the protective response. Although it was unclear whether T cell responses were generated in vaccinated animals, induction of IgG responses in vaccine recipients supports the possibility that the hexavalent vaccine might have induced virus-reactive T cells, as their help is critical for isotype switching. 

### 3.3. Recombinant Subunit Vaccines

Recombinant vaccines are routinely developed against infectious diseases, but only a few have been developed against CVB3. The VP1 protein has been used as a target to develop CVB3 recombinant vaccines. One study engineered a split intein from the *dnaB* gene of *Rhodothermus marinus* (*Rma*), termed *Rma DnaB* intein, which created a split functional N- and C-terminal intein to cyclize the VP1 protein of CVB3 in *Escherichia coli* (*E. coli*) [134]. This vaccine provided moderate efficacy, as only 60% of the mice survived, and mild myocarditis was present, despite generating VP1-specific IgG Abs and IFN-γ^+^ T cell responses [134] (Table 1). Further studies using a mucosal chitosan platform to incorporate a bi-functional protein from *E. coli*, termed FimH, revealed M-cell targeting and toll-like receptor 4-agonistic properties that also acted as an adjuvant was tested as a vaccine candidate [135] (Table 1). The vaccine responses included the generation of VP1-specific nAbs and T cell responses in mesenteric lymph nodes. However, the vaccine failed to provide complete protection, as only 60% of animals survived, and mild myocarditis and viral loads were still present [135]. More recently, a streptococcal protein-G derived, draining lymph node-targeting albumin-binding domain (ABD) peptide fused with the VP1-protein vaccine was tested in Balb/c mice. The data revealed ~73% survival rates, but mild myocarditis and viral loads were still evident. However, the vaccine induced the generation of IFN-γ^+^ CD8^+^ T cells in draining lymph nodes with enhanced CTL responses [136] (Table 1). An attempt was made to derive soluble VP1 constructed using adenovirus that induced nAbs and CTL response, but the vaccine efficacy was not tested [137]. Moderate efficacies of the vaccines described above may mean that the inclusion of other structural proteins is necessary to design subunit vaccines. In that direction, immunostimulating complex technology was used to incorporate all the structural proteins of CVB3 (VP1 to VP4). The immunostimulating complex produced high nAbs, and the challenged animals did not develop myocarditis, but the ability to prevent pancreatitis was not tested [125,138]. More recently, tag-free VP1 inclusion body nanoparticles were produced using the truncated *Ssp DnaX* mini-intein spontaneous C-cleavage system in *E. coli*. Oral administration of these inclusion bodies induced mucosal immune responses and the vaccine recipients were protected from CVB3 myocarditis with a survival rate of 60% [139]. While such adjuvant-free platforms are interesting vaccine candidates, their ability to prevent infection in other organs, especially the pancreas, is critical, since CVBs generally induce pancreatitis.

### 3.4. Vector Vaccines

Recombinant viral vectors have been used since the late 1980s to deliver foreign genes, with the most common being the Ad vectors. One study reported the development of a vesicular stomatitis virus vector expressing CVB3 VP1 (Table 1), and intranasal administration of this vaccine led to the protection of 70% of animals against a lethal challenge of CVB3, and the vaccine induced high nAbs and CMI responses [140]. Likewise, recombinant CVB3 expressing IFN-γ led to protection against myocarditis, as well as pancreatitis [141]. However, one major disadvantage of the use of vector vaccines is the generation of vector-reactive antibodies that act as a barrier to inducing memory responses upon booster vaccinations. While this limitation can be overcome with a prime-and-boost strategy with a recombinant viral protein, the lack of well-characterized immunogenic proteins may further impede the progress of vector vaccines. Alternatively, the use of vectors similar to those derived for severe acute respiratory syndrome coronavirus 2 (SARS-CoV-2) vaccines may help reduce the induction of vector-specific immune responses, but the occurrence of vector-borne side effects may occur in vaccine recipients [162].

### 3.5. DNA Vaccines

The DNA vaccination strategy was developed in the early 1980s when Enzo Paoletti and Dennis Panicali genetically engineered DNA from the cowpox virus and inserted genes from other viruses, such as herpes simplex, influenza, etc. [163]. Using this approach, a DNA vaccine was generated by cloning CVB3 VP1 DNA sequences into plasmid porcine cytomegalovirus and inoculating mice with the recombinant plasmid DNA [142,143] (Table 1). In this study, only 72.2% of the mice were protected from a lethal CVB3 challenge, with low levels of VP1-specific nAbs. Although IFN-γ and Interleukin-6 were elevated, mild myocarditis was evident in a few animals [142,143]. Likewise, a mucosal chitosan DNA vaccine platform targeting the nasal/oral tract was developed [144]. The initial development of the chitosan vaccine incorporated the CVB3 VP1 DNA sequence in the pcDNA3 vector encapsulated in chitosan (natural mucus adsorption enhancer), leading to the expression of recombinant CVB3-VP1 DNA in the mouse nasopharynx after intranasal inoculation [144]. The vaccine-induced specific secretory IgA, IgG, and CTLs conferred moderate protection (43%) of vaccinated mice. A virus was detected in the heart, and mild myocardial lesions were present [144]. To circumvent this problem, high-mobility group box 1 (HMGB1) and absent in melanoma 2 (AIM2) expression plasmids were co-transfected with VP1 plasmid, leading to the generation of recombinant chitosan VP1 vaccines [145,146,147]. Both HMGB1 and AIM2/chitosan VP1 vaccines enhanced sIgA and CTL responses and increased survival rates from 42 to 75%, leading to a decrease in the incidence of myocarditis (Table 1). 

Later, a novel approach to creating CVB3 DNA vaccines was investigated, whereby CVB3 VP1 DNA sequences were fused with either macrophage-derived chemokine (MDC), C3d3, Shiga toxin B subunit (STxB), or mouse beta-defensin-2 (mBD2) DNA sequences and inserted into the pcDNA3 vector [148,149,150]. Of these, pcDNA3/MDC-VP1 and pcDNA3/VP1-C3d3 induced better immune responses with reduced viral loads in blood, whereas enhanced CTL responses and Ab titers were noted with pcDNA3/STxB-VP1 and pcDNA3/mBD2-VP1 fusion vaccines [148]. Further studies with a DNA prime-protein boost vaccine regimen using rAd/MDC-VP1 and pcDNA3/MDC-VP1 vaccines revealed survival rates between 40–75% against a lethal CVB3 challenge, accompanied by enhanced immune responses and significantly reduced viral loads [149,150] (Table 1). Likewise, by constructing the DNA vaccines for CVB3 VP1 and VP3 proteins, the recombinant plasmids induced VP-reactive Abs but not the nAbs [151]. However, challenge studies revealed increased survival rates for the VP3 DNA vaccine relative to VP1 in challenge studies indicating that VP3-induced protection might have been contributed by T cell responses, but that was not investigated. Overall, although DNA vaccines could induce both antibody and cellular responses [164,165,166], their delivery platforms need to be optimized to induce effective responses. However, there have also been concerns regarding the insertion of foreign DNA into the host genome with the use of DNA vaccines in addition to a possibility of affecting genes controlling cell growth (activation of oncogenes or inactivation of tumor-suppressor genes) and development of tolerance or autoimmunity [167,168]. Nonetheless, reports suggest that the occurrence of random integration of plasmid DNA into the host genome appears to be low or negligible [169,170,171].

### 3.6. RNA Vaccines

The RNA vaccine approach to creating CVB3 vaccines has been adapted to address three expectations: (a) the mRNA encoding the vaccine expresses a full-length mutant CVB3 polyprotein; (b) it would undergo self-cleaving to generate mature viral proteins for antigen processing and presentation; and (c) it would not give rise to infectious virus particles. One study created a plasmid pH3IH1 encoding CVB3 with a mutated cleavage site between viral 2A and 3B proteins to avoid infectious particle generation, leading to deletions at the beginning of the 5′NTR region [152] (Table 1). The RNA vaccine could confer only 50% protection to immunized mice, and pancreatitis was evident in many animals. Another study utilized the lentiviral system to deliver short hairpin RNAs against sequences in the highly conserved *cis*-acting replication element within the 2C protein of CVB3, designated MET-2C *lenti*. This vaccine induced a reduction in viral titers, myocardial lesions, and pro-inflammatory cytokines, but with a survival rate of only 50% [153] (Table 1). 

### 3.7. Virus-like Particles (VLPs)

VLPs are similar in shape and size to a natural virus but lack genomic material (nucleic acids), and hence are non-infectious and do not revert to a virulent form by reversion, recombination, or re-assortment. VLPs are highly immunogenic and are expected to stimulate B and T cell immune responses [172,173,174]. Two VLP-based vaccines have been tested against CVB3 (Table 1). One of the initial studies used the entire coding sequence of the CVB3 genome (~6.6 kbs); it was cloned to create the pBlueBac4.5/cb3 vector [154]. Following a three-dose vaccination scheme, high nAbs were observed with no mortalities, but 90% of the mice had mild myocardial lesions and only one mouse was completely protected. Another study used a dual baculovirus transfer vector system, whereby one cassette included the P1 region of CVB3 and another included the whole genome except for the P1 region [155] (Table 1). The VLPs were produced in baculoviruses, and immunization of mice with these VLPs resulted in high CVB3-nAbs, but no challenge studies were performed to evaluate the vaccine’s efficacy. The VLP platform was further expanded to investigate immune responses to three enteroviruses: CVB1, norovirus, and rotavirus [156]. This combination of VLPs led to the production of high nAbs, IgG1, and IgG2a, but their use in challenge studies has not been reported. Likewise, VLPs produced in the baculovirus expression system were tested against CVB5 by injecting pregnant mice [157]. The passively transferred antibodies protected against CVB5 infection provide another strategy to prevent infections in newborns. Similarly, VLPs for CVB4 VP1 were also successfully generated in the baculovirus, but their potential use as vaccine candidates was not investigated [175]. Nonetheless, attempts have been made to improve the immunogenicity of VLPs by formalin inactivation that led to the induction of high nABs and virus-specific IgG1 response [158,159].

Overall, multiple approaches have been used to develop CVB vaccines (Table 1). A promising vaccine candidate is expected to elicit good B and T cell responses with no side effects. Live attenuated vaccines have been successful. However, the possibility of the attenuated viruses reverting to their pathogenic form cannot be excluded. Conversely, inactivated vaccines are safer than MLVs, but their inability to induce robust T cell responses is less than ideal for eliminating established virus infections. Alternatively, the generation of inactivated vaccines that induce high-affinity nAbs would have a significant impact on the prevention of CVB infections, given the lack of well-characterized immunogenic subunits with the CVBs to be able to produce effective vaccines by other formats such as subunits, nanoparticles, nucleic acids, and vector vaccines. 

## 4. Challenges and Future Perspectives

In contrast to other infectious diseases, vaccines for CVB infections have received little attention for many reasons. First, diseases caused by CVB are not severe enough to determine the immediate impact of CVB vaccines. However, from the perspective of myocarditis/DCM and T1D, the development of CVB vaccines may have merit. Still, their use is unlikely to have a dramatic effect on the occurrence of both diseases, since many non-CVBs and non-viral agents can trigger myocarditis and T1D [60]. Arguably, however, CVBs can still be the important triggers of both diseases [74,75,78,79,80,82,85,176,177]. Indeed, estimates indicate that the use of CVB vaccines can lessen the prevalence of T1D by at least 50% [176]. Second, determining the target population and age group for vaccinations may become contentious. While children represent the vulnerable population for viral myocarditis that can lead to congestive heart failure/DCM, evidence from the prototypic T1D model in non-obese diabetic mice suggests that exposure to CVB infection can delay the onset of T1D in young mice [178]. On the other hand, the same infections can aggravate T1D in adult mice with a possibility of developing anti-diabetogenic regulatory T cells [179,180,181]. These observations, however, may or may not be relevant to humans. Nonetheless, it is to be noted that the spontaneous development of T1D arising from genetic predisposition cannot be prevented by early exposure to CVB infections. Conversely, vaccinating young populations could mitigate the destruction of residual Islet β cells that would otherwise potentiate exposure to CVBs if left unvaccinated [182,183]. Third, the CVB vaccines should be safe to use. As they are RNA viruses, CVBs are prone to mutations and can revert to virulence as might occur with MLVs. Likewise, vaccines should be free of autoimmune responses, but such an event requires a well-established clinical disease to be able to induce tissue destruction in the heart [28,101]. The phenomenon of molecular mimicry has been proposed as an additional mechanism for the induction of autoimmunity to CVB. Such a possibility is unlikely at least for the generation of cross-reactive T cells due to a lack of significant homologies between CVB genomes and self-proteins [101]. However, the possibility of CVB infections indirectly inducing autoimmunity exists if, autoantibodies generated against one self-antigen cross-react with another as demonstrated with cardiac myosin [184,185]. Moreover, the data need to be cautiously interpreted because the mere appearance of autoantibodies does not necessarily mean that autoimmune reactions ensue. Nonetheless, major side effects of MLVs can be overcome by using inactivated vaccines that induce good nAbs. Fourth, it is not practical to develop vaccines for all six CVB serotypes individually since multiple serotypes can induce similar diseases. Therefore, a one-size-fits-all approach is needed. In that direction, polyvalent approaches, such as hexavalent and mRNA vaccines, are attractive candidates for development. Alternatively, monovalent vaccines that can induce cross-reactive protective responses against multiple serotypes, as demonstrated with the Mt10 vaccine virus, may have merit. Finally, the commercial viability can be a major barrier since the pharmaceutical industries may not come forward to market the CVB vaccines. This impediment can be overcome by emphasizing the need for CVB vaccines at a global level rather than focusing on selective geographical locations. Thus, to take the vaccines from bench to bedside, observations made in the preclinical models must be evaluated in non-human primate models and clinical trials. To date, only one clinical trial is currently in progress in Finland in relation to the use of a polyvalent whole virus-inactivated vaccine (ClinicalTrials.gov Identifier: NCT04690426). Any positive outcomes of this trial should boost efforts for developing similar vaccines that may lead to widespread acceptance.

The lessons learned from the use of vaccines against other RNA viruses, such as SARS-CoV-2, indicate that immune responses generated against vaccines may become irrelevant for escape mutants that may involve the phenomenon of original antigenic sin [186,187,188]. Such ineffective vaccine responses, especially non-neutralizing cross-reactive antibodies could potentially enhance the cellular uptake of virions in future encounters [188]. In these scenarios, it becomes a challenge to develop vaccines for newer variants, and obtaining continuous updates for their use may not be practical for CVBs because of the less severe nature of diseases induced by these viruses. Additionally, there may be a possibility that frequent administration of multiple boosters, as might occur with SARS-CoV-2 vaccines, may lead to immune exhaustion that may compromise the ability of naïve B cells to produce efficient and novel antibodies [188]. However, all factors considered, not using effective vaccines when available should not be an option for the public good. Perhaps we would still be living with smallpox today if the smallpox vaccine was not used. Thus, to be able to fight preventable infections, administrative efforts are required to create awareness in the public and to alleviate concerns and myths associated with the issues related to vaccine hesitancy.

## Figures and Tables

**Table 1:** Efficacy of vaccines used for various CVB serotypes.

Type	Approach	Model	Outcome	Ref
Live attenuated	CVB3: p14V-1 Mutation: D155G in VP1	SWR/Ola and Balb/c	Induced nAbs and low levels of anti-cardiac antibodies, but myocarditis was absent	[95,96,97,98]
	CVB3 (RD) Mutation: T151S of VP2	C3H/HeJ, C57BL/6, Balb/c, and C57BL/6-beige	Low nAbs and low viral titers in hearts and pancreata with pancreatic cell necrosis.	[102,103,104]
	CVB3 (KR/EG/DE) and (KR/EG/PM)	A/J	Low viral titers in the heart and generated nAbs; myocarditis was absent, but mild pancreatic damage was present	[105,106]
	CVB3 (H3) Mutations: Y240F and Y254F in the C-terminal region of VP2	Balb/c	Induced high nAbs and CTL response, but myocarditis and pancreatitis were absent	[107]
	CVB3 *Sabin3-like* Mutation: Stem-loop V (nt U475C)	Swiss Albinos	Induced low nAbs against CVB3 and CVB4-E2, but a few lesions were present in the heart and pancreas	[108,109]
	CVB3: Clinical isolates Mutation: Stem-loop II (nt 88–186)	C3H/HeJ and A/J	Low viral titers in the heart and pancreas; protected against CVB3 myocarditis	[106,110,111,112]
	CVB3/GA	C3H/HeJ	Protected against CVB3-induced myocarditis and pancreatitis	[113]
	CVB3: CPV/49 CVB3 5′NTR replaced with type 1 poliovirus	C3H/HeJ	nAbs against CVB3 and complete protection against both myocarditis and pancreatitis	[114]
	CVB3: Temperature-sensitive mutants	CD-1	Low viral titers in hearts, but myocarditis was absent; induced a low level of neutralizing IgG antibodies but low IFN-γ response	[115,116,117,118]
	CVB3-PL2-Ad2L1 CVB3/0 encoding Ad2 hexon L1 loop and protease 2A (2Apro)	Balb/c	Low nAbs against both CVB3 and Ad2, but myocarditis and pancreatitis were absent; high IgG1 titer	[119]
	CVB3: Incorporation of target sequences for miRNA-133 and miRNA-206 into the 5` UTR	Balb/c	High nAbs and survival rates, although mild tissue injury was seen in the heart and pancreas	[120,121]
	Mt-10 CVB3	A/J and NOD	High nAbs and virus-specific Ab and T cell responses; significant cross-reactive T cell and Ab responses against CVB1, CVB3, and CVB4; complete protection against CVB3-induced myocarditis and pancreatitis in A/J mice; significant protection (92%) against CVB4-induced myocarditis and pancreatitis in A/J mice; and significant protection (87%) against CVB4- accelerated T1D in NOD mice	[122,123,124]
Inactivated	CVB3: BPL treatment	Not reported	Low nAbs against CVB3 with a survival rate of 74%.	[125]
	Formalin-treated polyvalent CVB1 to CVB6	CD1 and New Zealand white rabbits	Low nAbs and viral titers in the pancreas, but no mortalities	[126,127]
	Formalin-treated CVB4-E2	NOD	Delayed onset of T1D, but enhanced antibodies to Islet β-cell auto-antigens	[128]
	Formalin-treated hexavalent vaccine (CVB1-6)	C57BL/6J, Balb/c, *SOCS-1*-Tg, and NOD Rhesus macaques	Strong nAbs; protection against acute CVB infections, CVB-induced myocarditis, and T1D Immunogenic and induced nAbs	[129,130]
	Formalin-treated CVB1 monovalent vaccine	*SOCS-1*-Tg and NOD	Strong nAbs; protection against CVB1 infection and CVB1-accelerated T1D	[129,130,131,132,133]
	Formalin-treated CVB4 monovalent vaccine	NOD	Moderate nAbs with complete protection against CVB4-accelerated T1D	[129]
Recombinant subunit	*Rma DnaB* intein cyclization of CVB3 VP1	Balb/c	Increased VP1-specific IgG, IFN-γ^+^ T cells with 60% survival rate but mild myocarditis and viral loads were detected in hearts	[134]
	CVB3: FimH-Chitosan-pVP1	Balb/c and C57BL/6	Increased sIgA and virus-reactive T cells with a 60% survival rate, but mild myocarditis and virus load were detected in hearts	[135]
	CVB3: ABD-VP1 fusion protein	Balb/c	A 73% survival rate with mild myocarditis and viral load was noted, accompanied by increased CTL and virus-specific memory T cell responses	[136]
	CVB3 sVP1-C3d3, constructed from recombinant Ads	Balb/c	Induced nAbs and CTL response	[137]
	CVB3: ISCOMs	Balb/c	High level of nAbs and all animals survived the challenge; complete protection against CVB3 infection	[125,138]
	Tag-free VP1 inclusion body nanoparticles	Balb/c	Increased mucosal response and moderate protection against CVB3 myocarditis	[139]
Vector	CVB3: rVSV-VP1	Balb/c	A 67% survival rate, reduced myocarditis and viral loads, and induced nAbs and CMI responses	[140]
	CVB3/IFN-γ	Balb/c	No tissue damage; no detectable virus; and no inflammation in the heart and pancreas	[141]
DNA	CVB3: pCMV/VP1	Balb/c	Reduced cardiomyocyte destruction, low nAbs, and elevated IFN-γ and IL-6 with a ~72% survival rate	[142,143]
	CVB3: Chitosan DNA vaccine/HMGB1/AIM2/LTN	Balb/c	Increased mucosal sIgA and IgG, CTL responses in spleens with a 42–75% survival rate and reduced myocarditis and viral loads	[144,145,146,147]
	CVB3: pcDNA3-STxB/C3d3/MDC/mBD2-VP1 and rAd/MDC-VP1	Balb/c	Increased nAbs and CTL response; and reduced viral load with a 40–75% survival rate, but mild myocarditis was noted	[148,149,150]
	CVB3: pCA-VP3, pCA-VP1	Balb/c	Detected VP-reactive Abs, but not nAbs; increased survival rates were noted with VP3 DNA	[151]
RNA	CVB3: pH3IH1	C57BL/6	A 50% survival rate; no infectious particles generated; and reduced viral titers, but pancreatitis was evident	[152]
	CVB3: MET-2C *lenti*	Balb/c	A 50% survival rate; no infectious particles were generated; and reduced viral titers, myocardial lesions, and pro-inflammatory cytokines	[153]
VLP	CVB3: pBlueBac4.5/cb3 expressed in Baculovirus expression system	SWR/J	A 100% survival rate with enhanced Ab responses, but mild myocarditis was detected in 90% of animals	[154]
	CVB3: Dual cassette pFastBac CVB3	Balb/c	No challenge studies were performed; but induced high nAbs	[155]
	Recombinant virus-derived nanoparticles from CVB1, norovirus, and rotavirus	Balb/c	Strong nAbs; IgG1 and IgG2a responses	[156]
	CVB5: Baculovirus expression system	Balb/c	Complete protection of suckling mice against CVB5	[157]
	Formalin-treated CVB1-VLP	Balb/c and C57BL/6	High nAbs and CVB1-specific IgG1 response	[158,159]
